# Methods of Generating Emotional Movements and Methods of Transmitting Behavioral Intentions: A Perspective on Human-Coexistence Robots

**DOI:** 10.3390/s22124587

**Published:** 2022-06-17

**Authors:** Takafumi Matsumaru

**Affiliations:** Graduate School of Information, Production and Systems (IPS), Waseda University, Kitakyushu 808-0135, Japan; matsumaru@waseda.jp

**Keywords:** human-coexistence robot, human-symbiotic robot, emotional movement, forthcoming movement, behavioral intention, embodiment, bodily movement, informative motion, preliminary motion, preparatory motion

## Abstract

The purpose of this paper is to introduce and discuss the following two functions that are considered to be important in human-coexistence robots and human-symbiotic robots: the method of generating emotional movements, and the method of transmitting behavioral intentions. The generation of emotional movements is to design the bodily movements of robots so that humans can feel specific emotions. Specifically, the application of Laban movement analysis, the development from the circumplex model of affect, and the imitation of human movements are discussed. However, a general technique has not yet been established to modify any robot movement so that it contains a specific emotion. The transmission of behavioral intentions is about allowing the surrounding humans to understand the behavioral intentions of robots. Specifically, informative motions in arm manipulation and the transmission of the movement intentions of robots are discussed. In the former, the target position in the reaching motion, the physical characteristics in the handover motion, and the landing distance in the throwing motion are examined, but there are still few research cases. In the latter, no groundbreaking method has been proposed that is fundamentally different from earlier studies. Further research and development are expected in the near future.

## 1. Introduction

The purpose of this paper is to introduce and discuss the following two functions that are considered to be important in human-coexistence robots and human-symbiotic robots: the method of generating emotional movements, and the method of transmitting behavioral intentions.

The most prominent feature of robots is that they have a body as an entity, and by moving that body, it is useful and beneficial to humans. Additionally, the main function of robots is to realize physical work by physical interaction with the environment using their bodily movements. In this paper, the function of transmitting emotional effects and behavioral intentions, i.e., the internal information of the robot, to humans using the bodily movements of robots is discussed. One solution to the problem of improving the affinity and friendliness felt by humans in human-coexistence robots is to design the robot movements so that humans can perceive the emotions and intentions of the robot.

In this paper, examples of major research and development are introduced and discussed. Here, we focus on when original ideas were first presented and new concepts were first proposed, regardless of whether they were published in academic journals or international conferences. Despite the important issues identified, we found that there are not many new methods, distinctive approaches, or interesting ideas. This also shows that the performance of the functions realized so far is still insufficient, that is, there is a lot of room for future research and development.

The rest of this paper is structured as follows: Section 2 deals with methods for generating emotional movements. It introduces not only studies on human estimations of emotions from robot bodily movements, but also studies on designing robot bodily movements so that humans can perceive specific emotions. Specifically, the application of LMA (Laban movement analysis), the development from the circumplex model of affect, and the imitation of human movements are discussed. Section 3 covers methods for transmitting behavioral intentions. It is not about the robot estimating the human status or intention, but about allowing the surrounding humans to understand the behavioral intentions of the robot. Specifically, the informative motions in arm manipulation (Section 3.2) and the transmission of the movement intention of robots (Section 3.3) are discussed. In Section 4, some messages are given as closing remarks.

## 2. Generation of Emotional Movements of Robots

There have been several reports of research trends on emotional expressions in the bodily movements of robots, such as [1,2]. However, many of them concern the recognition and classification of emotions received by an observing person, such as [3,4]. In contrast, this paper mainly discusses the generation of emotional movements in the robot body. That is, the methods by which emotion can be identified from the bodily movements of robots and also the methods by which the bodily movements of robots are processed so that some specific emotion can be recognized are examined.

### 2.1. Application of Laban Movement Analysis

In Laban movement analysis (LMA) [5,6,7], the representation components included in bodily movements are classified into the categories of Effort and Shape, and the bodily movements are evaluated based on them. The Effort component defines the dynamic characteristics and qualities of movement, and consists of four elements: Space (indirect (flexible)—direct), Weight (light—strong), Time (sustained—sudden (quick)), and Flow (free (fluent)—bound). The Shape component expresses the geometrical features of the whole shape, and is captured in three virtual planes: the Table (horizontal) plane, the Door (vertical) plane, and the Wheel (sagittal) plane. The LMA has been used for the analysis and evaluation of emotions that people can estimate from the bodily movements of robots.

Nakata et al. [8,9,10] proposed a “Labanian theory” that qualitatively describes bodily movements based on LMA. That is, they formulated the physical feature values based on LMA and applied them to six types of dance in a mammalian-type dance robot (a symmetric body with a head and two arms). By some experiments with participants, they confirmed the correlation between the calculated physical feature values and the emotions (joy, surprise (pleasure), sadness (grief), and anger) received by people from the bodily movements of the robot.

The EMOTE (Expressive Motion Engine) proposed by Chi et al. [11] is also a computational model of the Effort component and Shape component of LMA. The parameter adjustments shown for generating more natural synthetic gestures were subjective by manual settings. It has also been pointed out that it is important for the arm and the torso to work together for the generation of natural movements of animated characters.

Hachimura et al. [12] tried to extract motions corresponding to LMA components from a dataset of ballet dance performed by a skilled professional dancer obtained using a motion capture system. They formulated the feature values of Weight, Space, and Time, which are the elements of the Effort component, and extracted strong motions and weak motions in each element, respectively. The results were evaluated by comparison with the judgment of a LMA expert, and it was confirmed that the numerical formulation of LMA feature value is possible to some extent.

Barakova et al. [13] translated the Laban Effort parameters (Weight, Time, and Flow) to low-level movement parameters (curvature, velocity, and acceleration) based on the proposal by Rett et al. [14]. Proposing a framework for interpreting emotional movements, they designed behaviors of a small humanoid robot (NAO) to support group games for children to play. They pointed out the importance of different acceleration profiles of movement to design the emotional and social behaviors of robots.

Matsumaru [15,16] examined not only a method to identify emotions from bodily movements, but also a method to process bodily movements so that one specific emotion of four emotional options (joy, anger, sadness, or fear) could be put into the bodily movements of a small teddy bear robot with three links (right arm, left arm, and head) connected to its body with two-degree-of-freedom joints. The four kinds of emotion were set based on Pultchik’s wheel of emotions [17,18,19,20]. In this study, two standpoints were considered, a performer and an observer, to establish a dataset of emotional movements used in the analysis. Robot motion data were collected from the performer’s standpoint and were classified in the observer’s standpoint. First, fifty participants created the bodily movements of the robot with specific emotions. Then, the emotion discrimination in the collected motion data was carried out using the five feature quantities based on LMA; Weight effort (WE), Time effort (TE), Space effort (SE), Positional deflection (PD) in three directions, and Distance between parts (DP) on three planes, as in Nakata et al. [8,9,10]. The identification of the four emotions from the bodily movements was examined by comparing the result of the deviation score (DS) and the result of the discriminant analysis (DA), and the discrimination probability was around 60% in either method. When the motion data were identified using the principal components obtained by principal component analysis (PCA), the four emotions could be identified with a probability of about 70% or more. Furthermore, linear discriminant analysis (LDA) was used to clarify the characteristics of the principal components of each emotional movement. Then, in order to implement specific emotions in bodily movements, a method was proposed to process a basic movement based on the design principle settled from the characteristics of the principle component. Experiments with participants have shown that emotional movements can be generated so that people can interpret the intended emotions with a certain probability. Additionally, it was found that the movements with joy and anger were large, dynamic and frequent, so that intended emotion was easy to understand from the bodily movements. However, the movements with sadness and fear were small, static, and slow, so they were difficult to identify. It was also pointed out that the coordination and synchronization among nonverbal modalities (such as bodily movements and facial expressions) and the combination with auditory information (such as sound and voice) are important by a similar study on virtual characters [21] at around the same time.

Takahashi et al. [22] created movements with six kinds of emotions (joy, sadness, surprise, anger, fear, and disgust) based on Ekman’s basic emotions [23,24] proposed in conjunction with the classification of facial expressions. The same teddy bear robot as in [15,16] was used, also considering two major components (Effort and Shape) of LMA. The robot motion was created based on observations of human movements, so the design policy of emotional movements was qualitative and there was a lot of room for arbitrariness.

Samadani et al. [25,26] generated the opening and closing motion of (a) an anthropomorphic (human-like) hand model and (b) a non-anthropomorphic frond-like structure so as to recognize and generate emotional hand movements. A dataset was prepared which consisted of one movement type (opening and closing the hand) labeled in three emotional expressions (sadness, joy, and anger) by a demonstrator familiar with Laban notation. The FPCA (functional principal component analysis) was applied to extract features. The generated motion was evaluated by the LOOCV (leave-one-out cross validation) method using the one-nearest-neighbor misclassification error, and the relationship between the original emotional movement and the reproduced emotions were evaluated. It was shown that the original and regenerated affective movements were perceived quite similarly. This study focused on hand movements with remodeling and reproducing methods, but was not extended to apply to other parts of the body or other types of movements.

### 2.2. Development from the Circumplex Model of Affect

Many studies have referred the circumplex model of affect [27,28,29] proposed by Russell to estimate and implement emotions in bodily movements. This is a two-dimensional expression of emotion (affect): the positive/negative of valence (or pleasantness) and the high/low of arousal (or activation). Furthermore, Russell and Mehrabian have also proposed a PAD (pleasure–arousal–dominance) temperament (emotional state) model [30,31]. They argued that many emotions are captured in the three-dimensional coordinate system of PAD. Additionally, other emotions beyond pleasure, arousal, and dominance are arranged in the PAD space, such as exuberance (+P, +A, +D), hostility (−P, +A, +D), relaxation (+P, −A, +D), dependence (+P, +A, −D), docility (+P, −A, −D), anxiety (−P, +A, −D), disdain (−P, −A, +D), and boredom (−P, −A, −D).

Masuda et al. [32] examined four emotions (pleasure, anger, sadness, and relaxation) with reference to the circumplex model of affect. Specifically, they manipulated a small humanoid robot (KHR-2HV) to create emotional movements, set the Laban’s feature value set (Space, Time, Weight, Inclination, Height, and Area), and analyzed the correlation with the emotions estimated by participants. The equations to estimate the emotions contained in the whole-body movements were derived using multiple linear regression analysis. It was reported that the accuracy rate (the degree of agreement between the participants’ estimation and the equations’ estimation) was 85% or more.

Nakagawa et al. [33] generated emotional movements in a semi-humanoid robot, Robovie-mini, with four-degree-of-freedom arms and a three-degree-of-freedom head. Based on Russell’s circumplex model of affect, the valence level was associated with the basic posture of the robot, and arousal level was associated with the velocity and extension of joint movement of the robot. This method is used for conveying emotional nuances to users, and it would be applicable to any humanoid robot with various configurations in degrees of freedom. However, the basis for mapping emotions and movements was not fully examined. Additionally, this study did not aim to implement a specific emotion in the robot movements for certain tasks.

Glowinsk et al. [34] identified and analyzed the posture, shape and dynamic features of expressive gestures in emotion portrayals of human upper-body movements in a trial. They aimed to individuate the minimal representation of emotional displays based on nonverbal gesture features. They analyzed 120 emotion portrayals, in which 10 professional actors (5 females and 5 males) expressed 12 emotions as a subset of the GEMEP (Geneva multimodal emotion portrayals) corpus [35]. A set of 12 emotional states (elated joy/hot anger (rage), amusement/panic fear, pride/despair, pleasure/cold anger (irritation), relief/anxiety (worry), and interest/sadness (depression)) was created based on Russell’s circumplex model of affect. First, they identified that the four principal components of motion that express emotions were the activity of movement, the temporal and spatial excursion of movement, the symmetry of spatial range and posture, and the discontinuity and jerkiness of movement. By analyzing the relationship between these four principal components and the emotional states, it was suggested that an emotional behavior is distinguished into one emotional groups which is associated with one of four quadrants in the space of valence (positive/negative) and arousal (high/low).

Dael et al. [36] adopted the BAP (body, action, and posture) coding system to examine the description of emotional states by physical movements using a subset of the GEMEP corpus [35]. Specifically, they performed PCA (principal component analysis) on 49 behavior variables to extract 16 components, and analyzed the correlation between the 12 natural groupings (clusters) of emotion portrayals and the 12 emotion categories. Additionally, it was suggested that there may be few specific patterns for typical emotion in body posture, gestures, and movements.

Claret et al. [37] translated the emotional information which was expressed in the PAD (pleasure–arousal–dominance) space, based on the PAD temperament model, into kinematic features of motion in the JVG (jerkiness–activity–gaze) space. Then, using the JVG point and the position data of the user’s eye, the head orientation and body posture of the robot were calculated. Additionally, the motion velocity of the robot joints was generated by integrating the temporal sequence of hand poses to achieve the main task in the task priority inverse kinematics. This study particularly paid attention to the jerkiness (J) and activity (V) of the four principal components—energy (activity), spatial extent, smoothness/jerkiness, and symmetry (excursion)—which were extracted by Glowinski et al. [34] in the study on automatic feature extraction from expressive gestures. Based on the above-mentioned method, the movements (pleasure and arousal) of the arms and body and the amount of operation of the line of sight (dominance) of a semi-humanoid robot (Pepper, designed by Softbank Robotics) were determined and demonstrated. This method was later used as a general method for processing emotional states to be included in robot movements. It is effective for generating robot gestures and movements in a free space; however, some further ingenuity is required to extend it to movements in constrained spaces that involve contact with objects.

### 2.3. Imitation of Human Movement

There is also a method of generating emotional movements of a robot based directly on human motion data, or a database (DB).

Zecca et al. [38,39] created the whole-body movements of a humanoid robot KOBIAN that expressed emotions (anger, disgust, fear, sadness, confusion, happiness, and surprise) by human subjective manipulations. It was confirmed that people struggled to recognize emotions from only the whole-body movements of the robot, although it was also shown that the recognition rate could be improved when whole-body movements were accompanied with facial expressions. In addition, it was also reported that the whole-body movements created by a photographer and a cartoonist and the whole-body movements created by researchers were very different, and the emotions expressed by a professional actor were not always well recognized.

Kim et al. [40,41] proposed a generation method of robot motion based on a combination of contextual information and a behavior DB, aiming to realize a robot that provided an emotional interactive service. Specifically, human behavior data were collected in a DB, and a primitive DB was created from the behavior DB. The parameters (start and end positions, motor speed, time information, etc.) of the primitive DB were adjusted according to the context, and a linear interpolation method was applied. Then, 13 types of intentions (sentences) were roughly classified into 4 types—question (2), asking (2), response (4), and explanation (5)—with 3 types of emotional states (positive, neutral, and negative), so a total of 39 types of robot movements (facial expressions, neck motion, and arm gestures) were generated. A primitive questionnaire survey was conducted with 23 participants in which participants decided which one of five sentences was more appropriate for the presented motion expression: question (open question), asking (suggestion), response (approval), response (refuse), and informing (opening). It was reported that a motion without voice might have ambiguity, and the impression of motion expression was dependent on personal and subjective feelings.

Li et al. [42] investigated the recognition of emotions by participants from the created robot movements. They considered the six emotions (anger, disgust, fear, happiness, and sadness) based on Ekman’s basic emotions [23,24] in the same teddy bear robot as [15] and [22]. The difference in the perception by participants due to the difference between the provided and withheld context of the situation at the time of creating the robot motion and the difference in the perception by participants due to the robot motions created by puppeteers and laypeople (amateurs) were reported. However, the number of participants was limited, and the methods and guidelines to design robot gestures for the purpose of transmitting some forms of information were proposed as neither qualitative nor quantitative.

Erden [43] also studied the recognition of emotions by participants from movements of a small humanoid NAO, targeting three kinds of emotions (anger, sadness, and happiness) from six emotions based on Ekman’s basic emotions. The robot posture was determined based on the quantitative descriptions for a computer-generated mannequin [44], compiled from qualitative descriptions of emotional posture expressions in previous studies. It presented similar results to previous studies that there were still confusions mostly between anger and fear, happiness and surprise, and sadness and fear.

McColl et al. [45] generated body language in the upper body of a humanoid robot corresponding to a set of eight emotions (sadness, elated joy, anger, interest, fear, surprise, boredom, and happiness). They referenced the body language classification and the body movements and postures associated with specific emotions (body language descriptors) reported by Meijer [46] and Wallbott [47]. Similar results as in previous studies were presented, such as the importance of cooperation between body language, facial expressions, and vocal intonation.

Takahashi et al. [48] experimented with the influence of the emotional expression of a robot on human decision-making in a selection situation during the “Prisoner’s Dilemma Game” between a participant and a robot (small humanoid NAO). However, the emotional movements of the robot (joy, anger, shame, sadness (small), and sadness (large)) according to the selection situation were merely the reproduction of movements in which a researcher manually manipulated the robot limbs. In addition, the result that the participant acted more cooperatively when the humanoid robot acted more cooperatively rather than more competitively was similar to the findings in a previous study [49] (on an agent in the computer screen).

### 2.4. Behavior of Non-Biomimetic Robot

Human motion data are used for emotional expression not only in humanoid robots with different degrees of freedom, but also in robots with different structures that do not imitate living creatures (human beings, mammals, etc.) of animal size.

Karg et al. [50] attempted to generate emotional (pleasure, arousal, and dominance) movements of hexapods (six-legged robots) from a human emotional walking DB. They defined a “Froude number” that represented a dynamic similarity of walking styles, and used it to map different structures. From the experimental results, it was confirmed that several different levels of pleasure, arousal, and dominance were recognizable in the way the hexapod walked.

There are also reports of the relationship between the movements of non-biomimetic robots and the emotions that people can perceive.

Saerbeck et al. [51] used a mobile robot moving on a plane surface to examine the relationship between its movement and the emotions caused in people. They conducted a questionnaire survey, and the evaluation criteria were set based on the idea of the PANAS (Positive and Negative Affect Schedule) scale [52,53] and a SAM (Self-Assessment Manikin) [54,55]. The PANAS uses pairs (positive affect and negative affect) of 10-item PA (positive affect)/NA (negative affect) mood scales: interested/irritable, distressed/alert, excited/ashamed, upset/inspired, strong/nervous, guilty/determined, scared/attentive, hostile/jittery, enthusiastic/active, and proud/afraid. The SAM is an emotional assessment system devised to assess the three dimensions of pleasure, arousal, and dominance. The researchers recorded the interpretations of participants when the acceleration and curvature of the robot movement with the motion parameters were systematically changed. They found that the arousal level could be predicted from the acceleration, and that the combination of acceleration and curvature could affect the valence level. If a large amount of this kind of knowledge was systematically collected, it could be used as an elemental technique when designing robot movements that make people feel some emotions.

Knight et al. [56] investigated the interpretations of participants by changing the trajectory between the known start and stop positions of a mobile robot. They proposed a method to overlay the expressive motions of robots into actual actions for task execution. However, in reality, they only compared the impressions of participants on four types of changes in trajectory shape (line to line, sine to line, line to sine, and sine to sine) and obtained similar conclusions as on the impressions of human actions. If we can collect data under various conditions and clarify the relationship between the specific emotions felt by people and the trajectory shape of mobile robots, this may be applicable to the design of various robot movements.

### 2.5. Discussion

The above statements can be summarized in Table 1, Table 2, Table 3 and Table 4. It has been clarified that it is difficult not only to design robot movements so that humans can feel the intended emotion accurately, but also to make humans feel the intended emotion only from the robot movements. In other words, a general technique has not yet been established to modify any robot movement so that it contains a specific emotion in order to improve the affinity and friendliness that humans feel toward the robot. On the other hand, as already pointed out in various previous studies, consideration for consistency and synchronization in multi-modal information channels, including facial expressions and vocal sound (loudness, pitch, and speed), is important and effective for communication. Therefore, it is unnatural that only the movement is invariant when the robot expresses emotions, and it is significant that the movement is modified in various ways according to facial expression and vocal sound. There is still a lot of room for research and development on robot movements.

## 3. Transmission of the Behavioral Intention of Robots

The transmission of the behavioral intention of robots enables humans to read some physical information from robot movements, or enables humans to read the movement intention of the robot in some way. Section 3.1 introduces the matters related to this section as background information. Section 3.2 discusses research examples of informative motions in the arm manipulation of robots. Section 3.3 examines research cases concerning the transmission of the movement intention of robots. Section 3.4 shows research and development on the display function of the movement intention of a vehicle in self-driving cars. Additionally, in Section 3.5, discussions will be made based on these introduced cases.

### 3.1. Related Matters

Before getting into the main subject, several matters related to this topic need to be introduced.

#### 3.1.1. Attention to Preparatory Motion and the Preliminary Motion of Humans

The conscious or unconscious movements of a part of the body immediately before performing a task are called preparatory motion, preliminary motion, precursor motion, leading motion, predictive motion, and so on. It is considered that the information of the subsequent motion can be read from the preparatory motion, and the preparatory motion can be designed to inform some information. The importance of preparatory motions and preliminary motions was firstly pointed out in fields such as sports, animation, and games.

Ae et al. [57] filmed and investigated preparatory motion during the last stride of a Fosbury Flop by two high-speed cameras. They clarified the characteristics related to the decrease in approach speed and the large and effective takeoff motion.

Reitsma et al. [58] indicated that, in creating animations, it might be important for the sensitivity of viewers for the ballistic trajectory to be able to observe preparatory motions before the bullet firing.

Shiraki et al. [59] investigated the effect of preparatory motion on dart throwing and suggested that rhythmic preparatory motion leads to a better performance.

#### 3.1.2. Human Understanding of Behavioral Intentions of Robots

The importance of notifying intention in advance before taking any action, and promptly notifying the result of any action is well known as a matter of course, especially in the care and support of the elderly and disabled. Robots are said to be a movable form of artificial intelligence with a physical body, and their form and appearance are roughly divided into the biological type, that resemble existing creatures, and the non-biological type that do not necessarily resemble a living thing, regardless of being conscious or having a head, hands, or feet. In any case, since robots are artificial objects, designing the behavioral intention of robots so that people can understand them is a problem. The following are related studies that consider preliminary movement.

Takayama et al. [60] conducted an experiment with an animation in which participants evaluated robot action in a human-coexistence environment. They showed that, when the robot presented the forethought action before the essential action (expressive part) and the reaction to the result of the essential action (success or failure), it made it easier for people to understand the internal thinking process of the robot. However, the “forethought” was not defined sufficiently, concretely, or clearly, and the method of designing and setting the action, which should depend on the work content and situation, was not specified.

Gielniak et al. [61] developed a generation algorithm of anticipatory motion in an upper-torso humanoid robot, Simon. Specifically, a motion graph [62] including transition points between motion clusters was created from human motion data, and the anticipatory motions were extracted and synthesized to the robot motion. The researchers explained that the presence of anticipatory motion allows people to perceive the robot’s intentions faster. However, the distinction between anticipatory motion and essential motion was not fully explained here, and the difference between motion with and without anticipatory motion was not clear. Furthermore, the study did not extend to the design method of anticipatory motion for any arbitrary essential motion.

Tanaka et al. [63] proposed a numerical model that dealt with uncertainty and the time constraints, especially in real-world environments, to examine the effect of preparatory motion on robot agility.

Wortham et al. [64] displayed the action selection mechanisms on a screen as a real-time control state in order to improve the transparency of autonomous robots by visualizing the internal state and decision-making processes.

#### 3.1.3. Modeling and Prediction of Human Behavior

Research on modeling and the prediction of human behavior using machine learning techniques is underway.

Martinez et al. [65] studied the modeling of human motion using a deep RNN (recurrent neural network) for the purpose of human motion prediction. As a qualitative long-term motion, the Human 3.6M (H3.6M) dataset (fifteen kinds of activities such as walking, eating, smoking, discussion, etc.) was examined. They described the necessity of large datasets to learn the shorter-term dynamics of human motion.

Barsoum et al. [66] worked on HP-GAN (human pose prediction generative adversarial networks) to learn the probability density function of future behaviors under the condition of previous behaviors to understand and predict human kinematics. They also proposed a motion-quality-assessment model which quantified the quality of non-deterministic predictions.

Chiu et al. [67] proposed a TP-RNN (triangular prism recurrent neural network) as a method for predicting human behavior in both the short term and the long term. The effect was verified using the Human 3.6M and Penn Action datasets.

Wu et al. [68] developed a training system of martial arts with mixed reality (CG presentation of predicted behaviors of the opponent), using real-time human behavior prediction (2D behavior estimation, 2D behavior prediction, and 3D behavior restoration) based on the deep learning of RGB images. Furthermore, in [69], a real-time prediction system for table tennis using a long-term and short-term attitude prediction network was constructed, and the landing point of a tennis serve was predicted before the ball was hit on a table.

Xu et al. [70] investigated the movements of lower limbs (button touch, kick, rotation-al jump, and left–right jump) from a static state that are difficult to predict because they can be performed without a large movement of the center of gravity. They reported the existence of preliminary motion in the movements of the lower limbs and the predictability of significant motion from their observations. This study is highly acclaimed because of the implicit assumption that preliminary motions occur before significant motions in previous studies was strictly examined.

Most of the predictions of human movements by the machine learning technique so far are mainly predictions of types of movements, and the estimation of the degree of movements may be limited, for example, to the discrimination of strength and weakness.

#### 3.1.4. Information Transmission by Projection

A method using projection in collaborative work between a user and a robot has been proposed for about 20 years in order to efficiently transmit robot intentions of operation to a user.

Wakita et al. [71] tried to intuitively relate work content and robot motion by projecting the information about the work to be performed by the robot on a floor surface or wall surface.

Machino et al. [72] proposed SCOPE (sight collaboration by projection effect) using a set of cameras and a projector mounted on a mobile robot for remote collaboration between workers with a shared field of view.

Lee et al. [73,74] proposed a “ubiquitous display” as a main device to realize an “intelligent space”, and developed a mobile robot that projects signs and maps (as daily information sources) on floor or walls when giving directions to a user.

Kirby et al. [75] pointed out that since robots are artificial objects, human social conventions would be constraints on robot guidance.

### 3.2. Informative Motion in Manipulation

Movements and behaviors that include non-explicit information are called “informative motions” [76]. The informative motions in arm manipulation include ones that transmit some information during motion to perform a physical task (such as a reaching motion or a handover motion), and others that convey some information by the motion (preparatory motion or preliminary motion) just before the motion to execute the task (such as a throwing motion).

#### 3.2.1. Design of the Reaching Motion of Robots

The reaching motion is the motion of extending the arm toward the target and bringing the hand to the target position. The following studies are specialized in the reaching motions of robots and aimed to design motions to be understood by people.

Dragan et al. [77] insisted that the “predictability” (matching what is expected) of a robot movement is fundamentally different from the “legibility” (expressing the intent of movement). They explained this with a simple reaching task, proposed a mathematical model, and showed a planning method of robot trajectory based on cost optimization. However, they only examined the situation in which there was a possibility of either of the two as a goal-oriented operation. Therefore, in [78], the researchers conducted experiments with participants where targets were increased to four, and the one from three preset trajectories was selected, including obstacle avoidance. Additionally, similar results have been obtained in other studies.

Stulp et al. [79] argued that, in order for people to quickly and reliably predict robot intentions, it is necessary for the robot to exhibit legible movements to people. They attempted a stochastic optimization of policies using PIBB (policy improvement through black-box optimization) in the unsupervised reinforcement learning for the purpose of the rapid and normal completion of collaborative tasks between a user and a robot. Specifically, they showed a state to acquire a motion that was easy for the user to understand by repeating the learning in the trajectory generation with the robot to reach the goal based on the reaction time of users and the correct answer rate. However, they admitted that the learning method had the limitation that the other tasks required policy re-optimization accordingly.

#### 3.2.2. Design of the Handover Motion of Robots

The handover motion is the motion of handing an object from one’s own hand to the other’s hand. The following studies focus on the design of human-understandable motions, specializing in the handover motions of robots (summarized in Table 5).

1. Adding information about an object;

Matsumaru [80] investigated the whole-body movement of a humanoid robot that informed the recipient of the weight of the handover object. He measured and analyzed the movement of the human body that performs the handover task between people in order to clarify the features of the change in movement due to the weight of the object. To investigate whether people can understand the difference in the weight of objects, an experiment with participants was conducted in which the robot movement designed based on the extracted features was presented in a simulator. It was found that it was difficult for human recipients to estimate the exact weight of the object, but that they could judge whether it was heavy or not before receiving it.

2. Position accuracy, working speed, and synchronization;

Koene et al. [81] investigated the relationship between the spatial and temporal accuracy of a robot and the human satisfaction with the handover task between the user and the robot, assuming that the user (an automobile mechanic) was working in three different postures. It was clarified that the user emphasized the arrival time rather than the accuracy of the arrival position of the handover object.

Kshirsagar et al. [82] have proposed a controller that operates by STL (signal temporal logic) [83] to compensate for the timing of each stage in the handover task by a robot.

3. Object grasping;

Aleotti et al. [84] proposed the “grip affordance” concept. They insisted that when passing an object from a robot to a recipient, the robot not only considers the delivery position and the size and posture of the recipient, but also grips the part of the object excluding the part that the recipient will grasp, while the “algorithm for part-based grasp synthesis” [85] is applied for the way of grasping the object by the robot. The design policy is reasonable so that the robot can present an object to the recipient in consideration of not only the position and orientation of the object in space but also the part of the object and method of grasping used by the recipient. However, a method making it easy for the recipient to grasp the object using his or her hand was not considered in this study.

Chan et al. [86] observed the handover motion from a user to a robot to obtain the “handover configuration” (position and direction of grasping) of the user, and they created a humanoid movement to reproduce it. In [87] and [88], they propose the “affordance axes” associated with the object from the observation results of human-to-human handover. They suggested the possibility of applying it to teach the robot a natural and appropriate way of handover. This suggestion seems important, although the affordance axes should be automatically acquired from the shape of the object and the arrangement of the human fingers, and the way to grasp an object by a recipient depends on the orientation of the object and the robot trajectory to present it.

4. Handover position;

Suay et al. [89] applied the collected data of the angles and torques of the joints during the human-to-human demonstration to a biomechanical model. They proposed an algorithm, including parameter search, filtering, and optimization functions, to generate an optimal handover position.

Parastegari et al. [90] applied the human-to-human demonstration data to an ergonomic joint torque model and used the DJT (dyadic joint torque) cost function to predict the optimal transfer position (height and distance).

5. How to release.

Han et al. [91] compared the different modes of releasing an object in the handover motion from a robot to a recipient. Specifically, three types were examined: the rigid release (after the robot fully extends its arm, then it releases the object when the hand is pulled), the passive release (the robot releases the object when the tensile force exceeds the set threshold, even while the robot is extending the arm), and the proactive release (the robot releases the object according to the pattern changes of the tensile force, even while the robot is extending the arm). These were evaluated by the subjective evaluation of participants and the completion time, and it was shown that the proactive release was extremely popular.

**Table 5:** Design studies on the handover motion of robots.

Item	Study
Adding information about object (weight)	[80] (2009)
Position accuracy, working speed, and synchronization	[81] (2014) [82] (2019)
Object grasping	[84] (2014) [86,87,88] (2015)
Handover position	[89] (2015) [90] (2017)
How to release	[91] (2019)

#### 3.2.3. Design of the Throwing Motion of Robots

The throwing motion is the motion of throwing an object far away using one’s own hand without using any tools. The following studies focus on the throwing motion of robots and aimed to design motions that people can understand (summarized in Table 6).

1. Adding information about object;

Matsumaru [92] examined the throwing motion to inform a recipient of the landing distance of the object. He measured and analyzed the human-to-human throwing motion to clarify the features of the motion variables with respect to the landing distance of the object. To experiment whether a recipient could predict the landing distance of the object, a humanoid animation was designed so that the extracted features were implemented. This focused on the backswing as a preliminary motion.

2. Generation of the throwing motion;

Lombai et al. [93] optimized the parameters of the discrete-time linear controller as a nonlinear optimization problem considering the maximum power of each joint in a 6-DOF rigid arm.

Yedeg et al. [94] showed that the backswing had a great effect on the reaching distance of an object because it could prolong the acceleration period of the object.

3. Learning the throwing motion.

Mulling et al. [95] proposed the MoMP (mixture of motor primitives) framework as a way to coordinate multiple skills so that the coordinated movements can be learnt from the physical interactions between people and robots. They realized a table tennis game coordinated between a human and a robot (including acquisition and throwing).

Zeng et al. [96] proposed a method of estimating and learning parameters such as object perception (using RGB-D image), grasping method, and throwing speed while training by trial and error in order to realize the throwing of an arbitrary object. They named it “Residual physics”.

### 3.3. Transmission of the Movement Intention of Robots

Robots that transport objects are expanding from being used as AGVs in limited spaces such as manufacturing factories and storage warehouses to being used in environments that coexist with ordinary humans. That is, the application of mobile robots has been tried and realized for transporting goods and monitoring patrols in facilities such as hospitals, hotels, and restaurants, the delivery of mail and luggage by moving on public roads, agent activities for cleaning and communication in general households, and so on. These mobile robots are equipped with various safety functions to avoid contact and collision with humans. That includes the ability to avoid a human or stop a robot when the robot notices a human approaching, the installation of a flexible cover that does not make the human feel pain, even if it comes into contact with the robot, and the function to make the driving part of a robot flexible and adjustable with hardware technology and software technology.

On the other hand, there is also a solution of adding a warning function to inform the surrounding people of the future behavior of the robot before it moves in a certain way. Since robots are artificial objects, it is difficult for humans to predict their movement intentions from their appearance without special consideration. Many conventional mobile machines simply blink, rotate a warning light, or play a melody from a loudspeaker to simply notify their approach. In particular, for large vehicles traveling on general roads, when changing direction at an intersection, voices and warning lights are used to alert people around to prevent accidents. However, by these methods, robots can simply send out rough behavioral information and only expect an unspecified number of people around them to move away from their planned route. In this case, the content of the information is simple and insufficient for the transmission of movement intention. When a robot transmits information to humans, it may use new means not limited to the same methods as humans, but these means must be easy to predict and understand from the general common sense of humans. In the following, the methods shown in Table 7 are introduced and discussed for the transmission of movement intentions (speed of movement and direction of movement) from robots to humans.

#### 3.3.1. Transmission of Movement Intention by a Lamp

A method of expressing the movement intention of a mobile robot by a lamp has been proposed. Lamps are familiar to ordinary people, such as the direction indicator lamps (blinkers) of passenger cars, and the rotating lights of AGVs (automated guided vehicles).

Matsumaru et al. [97] proposed lighting and arrows for notifying the forthcoming movements and movement intentions (speed and direction) of mobile robots. The effect of advance notice and the optimum timing were examined by simulation experiments of random movements (speed) along a straight line. In [98], the movement was extended to random movements (speed and direction) on a plane, and a similar study was conducted.

Muramatsu et al. [99] studied a combination of three rotating lamps in three colors (red, yellow, blue) mounted on the top of an outdoor mobile robot, and also examined the timing of lighting.

Kannan et al. [100] compared and examined the use of display characters, display graphics, and lamps (left/right) as external interfaces for delivery robots, either individually or in combination.

#### 3.3.2. Transmission of Movement Intention by Gaze (Line of Sight)

A method of expressing the movement intention of a mobile robot by gaze (line of sight) has been proposed. Gazing is a daily activity among people and is regarded as a social cue for nonverbal communication.

Matsumaru et al. [101] developed a mobile robot equipped with an “eyeball” that expresses the moving speed by the degree of opening and closing of the eyeball and the moving direction by the position of both the eyeball and the pupil. They also conducted a questionnaire survey comparing four types of eyeball expressions: a one-eyeball type, a two-eyeball type, a human ball (will-o’-wisp) type, and an armor-held type.

Lu et al. [102] aimed to determine the efficient passing of people and robots, and so they developed a route generation algorithm based on a cost map according to the detected human position, and they implemented the eye contact function as a social signal to inform the detected human of the mobile robot.

Yamashita et al. [103] developed a mobile robot with a face to inform approaching pedestrians of (1) noticing pedestrians (raising its face) and (2) moving directions for avoidance actions (turning its face). Turning your face is almost equivalent to turning your gaze.

#### 3.3.3. Transmission of Movement Intention by an Arrow

A method of displaying the movement intention of a mobile robot with an arrow has been proposed. An arrow is a familiar expression to ordinary people, such as on traffic signs.

Matsumaru [104] mounted a display on a mobile robot in which a displayed arrow expressed the speed of movement (in three stages) by the size (length and width) and color (in three colors, similar to traffic signals), and the direction of movement by the degree of its curvature. A questionnaire survey was conducted in comparison with the eyeball [101]. It was clarified that the arrow was preferred because it was a direct expression rather than a symbolic one, such as an eyeball.

Coovert et al. [105] projected an arrow with varying lengths and thicknesses and a simplified map on a given traveling surface to signify the short-term, medium-term, and long-term intentions of movement of a mobile robot.

Shrestha et al. [106,107] compared the display and projection of an arrow with the direction indicator lamps to inform the directional intention of a mobile robot. They reported that the projection of an arrow was evaluated as user-friendly and intuitive.

Huy et al. [108] developed a laser writer (a combination of an RGB laser and an XY-axis galvanometer mirror) instead of a projector so that it could be applied both indoors and outdoors, and they drew arrows and letters on the traveling surface.

Chadalavada et al. [109] compared the resident arrow, the blinking arrow, and the projection of planned paths as a method of communicating future orbital intentions of AGVs to people. They reported that the resident arrow was preferred.

Hetherington et al. [110] compared the blinking light, the projected arrow for the direction of the target, and the projected arrow for the direction of movement as cues to make the movements of a mobile robot easier to understand. They recommended the projected arrow (direction of movement) as a socially acceptable signal.

#### 3.3.4. Announcement of Route and Area of Mobile Robots

A method has been proposed in which the movement intention of a mobile robot is displayed (irradiated or projected) on a given traveling surface as a planned route or an occupied area.

Matsumaru et al. [111] reported a mobile robot equipped with a device that draws a planned route on a traveling surface using a laser light source and a pan-tilt mechanism. They pointed out that the number of seconds to draw the planned route should be decided in consideration of the size and performance of the mobile robot. In [112], a projector was mounted on a mobile robot, and the planned route (including the speed of movement) in addition to the operating state (including the remaining battery) was projected on the traveling surface using symbols and characters. Furthermore, in [113], in order to compare and evaluate the four methods [98] proposed as ways of advance notice of the movement intentions of a mobile robot, the researchers conducted experiments with participants on the passing each other of a participant and the robot and the position prediction of the robot by participants. They concluded that, in order for people to respond quickly based on simple information, the method (eyeball and arrow) to announce the robot movement of just-after-the-present is preferable, and in order to avoid contact or collision based on more detailed and accurate information, the method (light ray and projection) to show the continuous robot movements from the present to a time in the near future is effective.

Chadalavada et al. [114] set a projector on a mobile robot and projected a green line indicating the planned route and a white line indicating the occupied width.

Watanabe et al. [115] mounted a projector on an electric wheelchair and projected a red belt indicating the planned route and occupied width.

#### 3.3.5. Information Transmission Using MR/AR Technology

Recently, the application of MR (mixed reality) and AR (augmented reality) to the transmission of the movement intention of robots has been studied. However, with this method, a person who wants to obtain the information needs to wear a special device, such as a HMD (head-mounted display).

Rosen et al. [116] presented the planned movement of a robotic arm to people by MR visualization on a HMD. Through evaluation experiments, they indicated that this approach could transmit the trajectory information from a robot to a user more quickly and accurately than the 2D display/mouse with 3D model visualization.

Walker et al. [117] used AR-HMD to present the three-dimensional flight path of a drone to a user. Four types of display methods were prepared: (1) NavPoint: waypoints + a moving sphere for the speed of movement and the arrival/departure timing; (2) arrow: an arrow moving in space for the route/speed of movement and the arrival/departure timing; (3) line of sight: an eyeball looking at the destination; (4) utility: 2D radar data + texts in an information box + indicators. They conducted an experiment comparing these display methods and reported that NavPoint was superior.

**Table 7:** Transmission of the movement intentions of robots.

Method	Study
Lamp	[97,98] (2001, 2003) [99] (2016) [100] (2021)
Gaze (line-of-sight)	[101] (2005) [102] (2013) [103] (2019)
Arrow	[104] (2007) [105] (2014) [106,107] (2016, 2018) [108] (2017) [109] (2020) [110] (2021)
Projection	[111,112,113] (2006, 2008) [114] (2015) [115] (2015)
MR/AR	[117] (2018)

### 3.4. Display of Recognition and Intention of Self-Driving Cars

Vehicles that autonomously recognize, judge, and operate, such as self-driving cars and unmanned ground vehicles, are becoming a reality. There is a sense of urgency and a sense of crisis in the industry in the development and practical application of the function of recognizing the environment and situation by such a vehicle, and presenting the movement intention of the vehicle. The major difference between self-driving cars and mobile robots is the size and speed of the moving object. It should be noted that the technology researched and developed in the field of robotics may not always be applicable to self-driving cars. For self-driving cars, methods of improving the environment and preparing large-scale equipment (for example, [118]) are also required. On the other hand, some research and development examples of display devices mounted on vehicles are introduced below.

#### 3.4.1. Information Transmission by LED Strings and Signs

Correa et al. [119,120] examined the transmission of the current state and movement intention of vehicles as one of the multi-modal interactions between an autonomous forklift and nearby users. They implemented an LED string (with moving light patterns such as chasing) and an LED sign (character displays of “manual”, “active”, “call: storage”, and “collection: detection”).

Florentine et al. [121,122] placed an LED string around the body of an autonomous golf cart. LEDs in the direction in which there were no obstacles at a short distance were turned on in blue, and LEDs in the direction in which obstacles were detected were in red.

Habibovic et al. [123] installed an RGB-LED string with a length of 1 m on the upper part of the windshield of an autonomous vehicle as an AVIP (automated vehicle interaction principle). They studied a visual interface that displayed four types of signals to pedestrians and conducted experiments at the pedestrian crossings and the parking lots: (1) lighting only at the central part meaning “I’m in automated mode”; (2) lighting expanding laterally from the center meaning “I’m about to yield”; (3) slow pulsation from complete lighting meaning “I’m waiting”; and (4) lighting shrinking toward the center meaning “I’m about to start driving”. Furthermore, in [124], an online survey was conducted on the combination of five colors and three types of animation patterns of the light band installed on the bumper of the vehicle. It was reported that evenly blinking or pulsating animation was preferred.

#### 3.4.2. Information Transmission by Projection

Ochiai et al. [125] equipped two types of output devices to extend driver functions for human-to-human communication in a passenger car: (1) robotic eyes moving together with the eyes of the driver; and (2) a projector that projects the hand sign by the driver.

Mercedes-Benz [126] announced a concept car for self-driving, F 015 Luxury in Motion, which has a large front and rear LED display and a front-facing laser projection system for visual communication with pedestrians. On the front LED display, an undulating light sequence is displayed to indicate “standby”, and an unexpected stop is indicated by the letters “STOP”. The laser projection system projects important information, such as a virtual pedestrian crossing, on the road surface ahead.

Mitsubishi Electric [127] presented a system that projects the information of front and rear paths, door openings, and emergency stops to pedestrians and drivers of other vehicles on the road surface.

#### 3.4.3. Comparative Experiment of Displays about Recognition and Intention

Clercq et al. [128] investigated the effect of the eHMI (external human–machine interface) of an approaching vehicle on the intention of pedestrians crossing the road by virtual reality experiments using an HMD. The five types compared were: (1) no display; (2) brake lamp; (3) knight rider animation (lighting part moves when decelerating); (4) smiley (downward convex single curve); and (5) character (“don’t walk” or “walk”). It was shown that almost all interfaces required learning, but only characters could be understood directly.

### 3.5. Discussion

In the first half of Section 3, several research cases on informative motions in the arm manipulation of robots were introduced. In particular, it is a challenging issue to design robot movements to include some physical information. As introduced here, the target position of the hand in the reaching motion, the physical characteristics of the object in the handover motion, and the landing distance of the object in the throwing motion have been examined, but there are still few research cases. Machine learning based on big data of human movements may be able to generate such robot movements by inputting values to some parameters, but that is not interesting. By systematizing various related knowledge such as cognitive psychology, social psychology, morphogenesis, and biological behavior, and by compiling common knowledge, implicit knowledge, and empirical knowledge among humans, we may be closer to revealing the mechanisms of human cognition regarding motions and movements, and this may be applicable to the design of robot movements. There may be hints, for example, in the feints in competitive sports, the body manipulations in martial arts (reading: sensing the opponent’s willingness to attack in advance; marking: observing the opponent for successful reading), the movements in pantomimes to express concrete things, and the movements in other stage performing arts such as ballet and dance to show abstract things.

In the latter half of Section 3, research and development examples related to the transmission of movement intentions of robots were introduced. The authors [129] proposed methods to present forthcoming movements as visual information. These were categorized into two types: (1) an advance notice of the state at a certain time in the future; and (2) an advance notice of continuous movements from the present to a certain time in the future. Concretely, there were four methods: (a) lamp; (b) party horn (expandable arrow); (c) ray; and (d) projection. The former two were type (1), and the latter two were type (2). These four methods were embodied as (a) eyeball (the direction of movement by the eyeball position, and the speed of movement by the eyeball size); (b) arrow (the direction of movement by arrow direction, and the speed of movement by arrow length); (c) light-ray (drawing the planned route up to a certain time on the running surface); and (d) projection (drawing the occupied range up to a certain time on the running surface). The effect was verified by experiments, and the characteristics of each method were clarified [130,131]. No groundbreaking method has been proposed that is fundamentally different from these earlier studies. It is expected that new methods will be proposed by future research and development.

## 4. Remarks

Techniques that promote communication even when there is a distance between people and robots will become more and more important for collaborative works and cooperative tasks between humans and robots under various constraints caused by the epidemic of the new coronavirus infection.

Observation power and imagination are necessary to come up with new research subjects. If you consider the necessary functions by assuming the situation in which the robot works in detail, you may come up with a good research subject. Even if you come up with an interesting research subject, some steps may be required before it can produce great results. At first, it should be modeled by software, and simulation experiments should be performed using it to produce some preliminary results. If the results can be announced at academic conferences and other opportunities, it may be possible to obtain larger research funds. If you have the money, you may be able to make hardware and conduct verification experiments in a mockup that is close to reality. Demonstrations using hardware are very important in the research and development of robots and mechatronics systems.

As far as the author knows, this paper focuses on where and when original and novel research and development works are presented. Of course, when conducting a literature survey to discuss previous works and related studies, it is necessary to confirm recent trends and technical levels. For example, in the field of image recognition, when you present your research results, you need to investigate recent results by others carefully and in detail. This is to make a strict numerical comparison between the result of the algorithm you propose and the result of the most advanced algorithm, for example, using the object detection accuracy as an evaluation index. On the other hand, in hardware-based research and development, after an original idea is announced and an innovative concept is proposed, even the latest research and development is often merely reworking or fine-tuning. Therefore, it is important to pay attention to and respect not only recent research and development but also the research and development works that first proposed and executed the original idea and innovative concept.

It is expected that the interest in the issues introduced and discussed here will increase, and that research and development will become even more active. The future of robotics and mechatronics is still expanding and is a promising area. The author is looking forward to your success.

## Figures and Tables

**Table 1:** Emotion models.

Model	Reference	Emotion
Pultchik’s wheel of emotions	[17,18,19,20]	Joy, anger, sadness, fear
Ekman’s basic emotions	[23,24]	Joy, sadness, surprise, anger, fear, disgust
Russell’s circumplex of affect	[27,28,29]	Positive/negative of valence (pleasantness), high/low of arousal (activation)

**Table 2:** Description of methods to mimic human behavior.

Method	Study
LMA (Laban movement analysis)	[8,9,10] (1996, 1998, 2002) [11] (2000) [12] (2005) [13] (2010) [15,16] (2009) [22] (2010)
BAP (Body action and posture) coding system	[36] (2012)
Body language descriptors	[45] (2014)

**Table 3:** Data analysis methods.

Method	Study
PCA (principal component analysis) + LDA (linear discrimination analysis)	[15,16] (2009)
FPCA (functional principal component analysis)	[25,26] (2011, 2013)

**Table 4:** Design methods for emotional movement.

Method	Study
Feature superimposition	[15,16] (2009) [56] (2016)
Mapping valence from level and arousal level to basic posture and joint velocity	[33] (2009)
Translation from emotional information in PDA space to kinematic features in JVG space	[37] (2017)
Imitation of human movement	[38,39] (2008, 2009) [40,41] (2009, 2010) [50] (2010) [42] (2011) [43] (2013) [45] (2014)

**Table 6:** Design studies on the throwing motion of robots.

Item	Study
Adding information about object (landing distance)	[92] (2013)
Generation of throwing motion	[93] (2009) [94] (2013)
Learning of throwing motion	[95] (2013) [96] (2020)

## Data Availability

Not applicable.
